# Landscape and training opportunities in artificial intelligence for biosciences in Latin America: the BiotrAIn project

**DOI:** 10.3389/frai.2026.1931300

**Published:** 2026-10-14

**Authors:** Jose Arturo Molina-Mora, Flavio Ezequiel Spetale, Tulio de Lima Campos, Pedro E. Romero, Lucas Inchausti, Adrian Gustavo Turjanski, Ignacio Alcántara, Maria Fernanda Ribeiro Dias, Carla V. Filippi, Matías Domínguez, Juan Bizzotto, Daniela Senra, J. Noé García-Chávez, Claudia Hoepfner, Cleidy Osorio-Mogollon, C. Paola Rangel-Chávez, Valeria Foronda, Alejandra Rougon-Cardoso, Nelly Selem-Mojica, Yesid Cuesta-Astroz, Cindy Aguilar-Bartels, Rebeca Campos-Sánchez, Lizzie Tchongwe Divala, Kim Tamara Gurwitz, Cath Brooksbank

**Affiliations:** 1 Centro de Investigación en Enfermedades Tropicales (CIET), Centro de investigación en Hematología y Trastornos Afines (CIHATA) and Facultad de Microbiología, Universidad de Costa Rica, San Jose, Costa Rica; 2 Bioinformatics, CIFASIS - CONICET/UNR, Santa Fe, Argentina; 3 Bioinformatics Core Facility, Aggeu Magalhães Institute - Fiocruz, Recife, Pernambuco, Brazil; 4 Facultad de Ciencias Biológicas, Universidad Nacional Mayor de San Marcos, Lima, Peru; 5 Laboratorio de Bioinformática, Departamento de Genómica, Instituto de Investigaciones Biológicas Clemente Estable, Montevideo, Uruguay; 6 Departamento de Química Biológica, Facultad de Ciencias Exactas y Naturales; IQUIBICEN-CONICET, Universidad de Buenos Aires, Buenos Aires, Argentina; 7 Unidad de Bioestadística, Departamento de Salud Pública, Facultad de Veterinaria, Universidad de la República, Montevideo, Uruguay; 8 NUPEM/UFRJ - Instituto de Biodiversidade e Sustentabilidade, Universidade Federal do Rio de Janeiro, Rio de Janeiro, Brazil; 9 Hospital Israelita Albert Einstein, São Paulo, Brazil; 10 Departamento de Biología Vegetal, Facultad de Agronomía, Universidad de la República, Montevideo, Uruguay; 11 EEA Pergamino, Instituto Nacional de Tecnología Agropecuaria, Buenos Aires, Argentina; 12 Departamento de Física, Facultad de Ciencias Exactas, Universidad Nacional de La Plata, Buenos Aires, Argentina; 13 Laboratory of Agrigenomic Sciences, Escuela Nacional de Estudios Superiores, Unidad León, Universidad Nacional Autónoma de México, Guanajuato, Mexico; 14 Faculty of Sciences and Technology, Center of Biotechnology, Universidad Mayor de San Simón, Cochabamba, Bolivia; 15 Faculty of Interdisciplinary Studies, GEMA Center for Genomics, Ecology and Environment, Universidad Mayor, Huechuraba, Chile; 16 Departamento de Biologia Celular e Molecular, Faculdade de Medicina de Ribeirão Preto, Universidade de São Paulo, São Paulo, Brazil; 17 División de Ciencias de la Vida, Departamento de Ingeniería Bioquímica/Posgrado en Biociencias, Tecnológico Nacional de México, Instituto Tecnológico Superior de Irapuato/Universidad de Guanajuato, Guanajuato, Mexico; 18 Biomathematics, Centro de Ciencias Matemáticas, Universidad Nacional Autónoma de México, Morelia, Michoacán, Mexico; 19 Escuela de Microbiología, Universidad de Antioquia, Medell, Colombia; 20 Centro de Investigación en Biología Celular y Molecular (CIBCM), Universidad de Costa Rica, San José, Costa Rica; 21 Training Team, EMBL's European Bioinformatics Institute (EMBL-EBI), Cambridgeshire, United Kingdom

**Keywords:** artificial intelligence, biosciences, BiotrAIn project, capacity building, curriculum, Latin America

## Abstract

Artificial Intelligence (AI) is redefining biosciences by enabling the analysis of complex, large-scale biological and medical data. Latin America has demonstrated remarkable potential through studies applying AI to biomedical sciences often using locally generated data and addressing regional needs. However, the integration of AI into research and healthcare in this region faces several structural challenges, including insufficient infrastructure, lack of specialized training, fragmented research systems, and continuously constrained funding. To overcome existing limitations, capacity building at the regional level is crucial. This includes developing localized AI training programs and the reinforcement of a collaborative research framework. Collaboration across institutions and countries, along with engagement from industry and government, is key to fostering an enabling environment for the responsible and effective use of AI. One promising response to these challenges is the BiotrAIn project, a partnership involving EMBL-EBI, the University of Costa Rica, and CABANAnet, with support from the Chan Zuckerberg Initiative. The project is structured around three pillars: knowledge exchange, curriculum development, and regional training. BiotrAIn exemplifies how strategically designed, regionally adapted programs can build AI capacity in biosciences and foster more equitable scientific development across Latin America. Strategic efforts like BiotrAIn serve as essential building blocks toward a more robust, ethical, and sustainable AI ecosystem in the region, while offering a model that could be adapted by other regions globally. Ultimately, with coordinated investment, inclusive policies, and collaborative networks, Latin America can position itself as a significant contributor to global AI-driven bioscience.

## 1 Introduction: artificial intelligence for biosciences in Latin America

Artificial Intelligence (AI) refers to a set of computational methods, algorithms, and systems—both software and hardware—designed to emulate human cognitive processes (Hanassab et al., 2024). Its main objective is to perform data processing and analysis tasks, often involving large-scale datasets, enabling the design of strategies to extract information, build models that learn from data, make predictions, and solve problems, thereby supporting decision-making (Muñoz Andrade, 2024). This capability has become especially relevant in the context of the digital and technological revolution of recent decades, characterized by the massive production of data across multiple domains of society, including the life sciences (Berisha et al., 2021; Drosou et al., 2017).

In biosciences, the development of high-throughput molecular technologies has led to an unprecedented availability of data derived from omics platforms, such as massively parallel sequencing (genomics and transcriptomics), mass spectrometry (proteomics and metabolomics), as well as from medical and environmental records, imaging, and other large-scale biological databases (Zhao et al., 2016). This data explosion has created a highly demanding analytical environment that can be supported using disruptive technologies (Drosou et al., 2017). AI is a promising solution to solving biological problems by extracting non-trivial information from multimodal and multidimensional data (Molina-Mora and Herrera-Hidalgo, 2025; Shultz and Fahlman, 2017).

Although AI technologies have been under development for decades and are not completely new (Howell et al., 2024; Lecun et al., 2015), their popularity surged after 2020 due to the widespread availability of AI-driven (particularly generative AI) tools and the rapid advancement of complex models across diverse fields of knowledge (Grzybowski et al., 2024). As illustrated in Figure 1, the advancement and application of artificial intelligence are largely driven by machine learning approaches (Gherman et al., 2023), which rely on computational models and algorithms capable of learning from data to perform tasks such as classification, clustering, and deep learning-based pattern recognition (Toosi et al., 2021).

**Figure 1:**
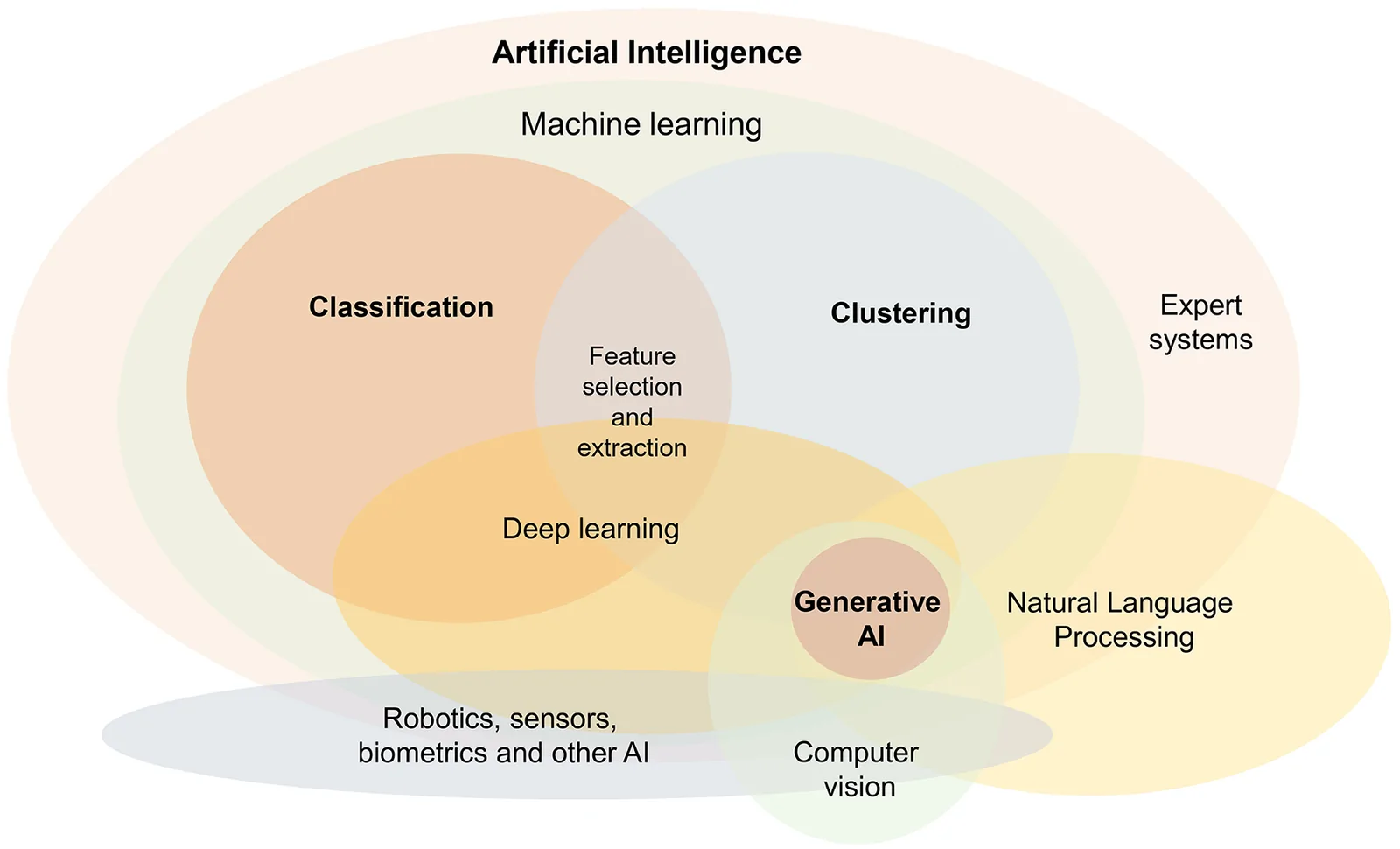
Overview of Artificial Intelligence, categories, subfields, and related areas.

These advanced AI approaches, combined with fields such as natural language processing (linked to linguistics for analyzing and generating human language) and computer vision (focused on object and pattern recognition) have enabled the emergence of generative AI models (Wong et al., 2024). In the scientific domain, one of the most transformative breakthroughs has been the development of deep learning models capable of predicting protein structures from amino acid sequences (Magaña et al., 2026), as exemplified by the development of AlphaFold (Jumper et al., 2021), as well as AlphaMissense (Cheng et al., 2023) or AlphaGenome (Avsec et al., 2026), that predicts the functional impact of coding and non-coding variants at genome-wide level.

While countries in the Global North (including the United States, Canada, the United Kingdom, members of the European Union, China, and Australia) exhibit high levels of preparedness for comprehensive AI adoption (Berisha et al., 2021; Gherman et al., 2023), much of the Global South—particularly Southeast Asia, Africa, and Latin America—continues to face substantial challenges to the systematic implementation of AI (International Monetary Fund, 2023). In particular, the region of Latin America—which consists of about 645 million inhabitants (8.37 percent of the global population), covers more than 19 million square kilometers, and is characterized by a vast ecological, ethnic, and social diversity—remains largely underrepresented in the global research ecosystem (Bolaño-Ortiz et al., 2020; Bottan et al., 2020; Leiva et al., 2023). The region struggles with economic and social inequalities, a fragmented research ecosystem, limited funding, and insufficient engagement with policymakers (International Monetary Fund, 2023; Molina-Mora and Herrera-Hidalgo, 2025). These structural barriers, coupled with the lack of shared infrastructure and coordinated research initiatives, continue to hinder scientific progress. Addressing these challenges calls for urgent investment in regional networking, cross-border collaboration, and enhanced capacities in research, development, and innovation.

Thus, the adoption of AI-based technologies in Latin America faces challenges, hindering the development in early stages. Various assessments, such as the AI Preparedness Index (AIPI), have revealed significant gaps in infrastructure, legislation, human resources, and ethical frameworks across the region (Berisha et al., 2021; Gherman et al., 2023; International Monetary Fund, 2023).

Despite these regional limitations, Latin America has made significant progress in applying AI across a variety of bioscience fields, including the study of pathogens, chronic human diseases, plant science, epidemiology, animal health, and automated image analysis (Alcántara et al., 2018; Campos et al., 2020a; Campos-Godínez et al., 2025; Castro-Castro et al., 2022; Chaves et al., 2025; Farias et al., 2024; Fariello et al., 2025; Fernández et al., 2021; Ferraz et al., 2025; Ferreira et al., 2020; García-Chávez et al., 2021; Gonzalez Dias Carvalho et al., 2023; Luoni et al., 2024; Martinez et al., 2022; Molina Mora et al., 2021; Molina-Mora et al., 2022; Molina-Mora and Herrera-Hidalgo, 2025; Morais et al., 2022; Mora-Poblete et al., 2023; Palande et al., 2025; Segura et al., 2017; Simon et al., 2022; Soares-Schanoski et al., 2019; Spetale et al., 2018, 2021). Some studies—either conducted using data from Latin America or developed through regionally driven strategies—are presented in Table 1. These examples demonstrate concrete efforts to expand the use of AI in the life sciences across the region. With improved access to resources, better talent retention mechanisms, stronger collaboration networks, a supportive regional research ecosystem, and appropriate legal frameworks, the development and implementation of AI-based models in Latin America hold great promise. This underscores the importance of continuing to build capacity at all levels and of promoting collaborative research networks in the biological and medical sciences throughout the region.

**Table 1:** Examples of studies in biosciences conducted in Latin America using AI strategies.

Country	AI approach	Field	References
Argentina	Clustering and Classification	Medicine (immuno oncology)	Fernández et al., 2021
Argentina	Classification	Imaging (agro-biotechnology)	Luoni et al., 2024
Argentina	Classification	Genomics (gene annotation)	Spetale et al., 2018
Argentina	Classification	Genomics (gene annotation)	Spetale et al., 2021
Brazil	Classification	Genomics (gene essentiality)	Campos et al., 2020b
Brazil	Classification	Genomics (gene essentiality)	Campos et al., 2020a
Brazil	Classification	Drug discovery	Ferraz et al., 2025
Brazil	Classification	Immunology	Gonzalez Dias Carvalho et al., 2023
Brazil	Classification	Plants (detection)	Ferreira et al., 2020
Brazil/Chile	Regression	Plant genomics	Mora-Poblete et al., 2023
Brazil/Mexico	Classification	Microbial regulatory genomics	Martinez et al., 2022
México/Brazil	Classification	Microbial regulatory genomics	Farias et al., 2024
Brazil	Classification	Immunology	Chaves et al., 2025
Brazil	Classification	Immunology	Soares-Schanoski et al., 2019
Brazil	Classification	Epidemiology	Araujo et al., 2022
Brazil	Classification	Digital pathology	Morais et al., 2022
Costa Rica	Clustering and Classification	Pathogen research (*Pseudomonas aeruginosa*)	Campos-Godínez et al., 2025; Molina Mora et al., 2021
Costa Rica	Clustering	Epidemiology (COVID-19)	Molina-Mora et al., 2022
Costa Rica	Clustering	Medicine (COVID-19)	Castro-Castro et al., 2022
Costa Rica	Clustering	Imaging (Cancer)	Molina-Mora et al., 2018
Mexico	Clustering and classification	Plants genomics (plant tissues classification)	Palande et al., 2025
Uruguay	Classification	Aquatic ecology	Segura et al., 2017
Uruguay	Regression	Aquatic microbiology	Alcántara et al., 2018
Uruguay	Regression	Aquatic ecology	Crisci et al., 2017
Uruguay	Regression (Transformers)	Genomics prediction	Fariello et al., 2025
Uruguay	Classification	Plants	Simon et al., 2022

In light of the numerous challenges facing the scientific development of AI and the research initiatives that are being consolidated in Latin America, EMBL's European Bioinformatics Institute (EMBL-EBI, https://www.ebi.ac.uk/), the University of Costa Rica (https://www.ucr.ac.cr/), and the CABANAnet network (https://cabana.network/) have launched the BiotrAIn project (https://www.ebi.ac.uk/training/our-partnerships/biotrain). This project, funded by the Chan Zuckerberg Initiative (https://chanzuckerberg.com/), aimed to strengthen regional capabilities in applying AI to bioscience research by developing a foundational, sustainable AI curriculum, leading to the use of this technology to better solve biological and biomedical problems.

## 2 The strategy: the BiotrAIn project

BiotrAIn builds upon the successful model implemented by CABANAnet, a program that strengthens regional capacities in high-throughput molecular technologies and bioinformatics for the study of pathogens, biodiversity, genomic surveillance, human genetics, and related areas, through research projects, secondment opportunities, training, and knowledge exchange activities (Campos-Sánchez et al., 2024). Thus, following a similar model, the BiotrAIn project promotes the use of advanced analytical tools for processing molecular, epidemiological, and biological data, with the overarching goal of contributing to a more equitable and participatory scientific ecosystem in the region.

The instruments, surveys and application forms used in this project were designed, developed, administered and disseminated by the core team (four members from the EMBL-EBI and three from the UCR/CABANAnet) within the framework of the project's structured capacity-building activities. Regular meetings were held throughout the project to define the objectives, target populations, instruments, and participant selection criteria, depending on the stage. Each designed task was subsequently discussed and refined with the trainer team before implementation.

The BiotrAIn project was developed in three main phases from 2024 to 2026:

### 2.1 Knowledge exchange meeting (KEM)

The KEM event on the use of AI in the biosciences across Latin America was held virtually in December 2024. Participation was based on interest and availability rather than a formal selection procedure. The event was disseminated through established professional and scientific networks by the CABANAnet project, University of Costa Rica and the EMBL-EBI, and social media as well. The project team also used regional contacts to encourage participation from different Latin American countries and scientific backgrounds. Thus, this event brought together more than 40 professionals working on machine learning (including classification, clustering, and deep learning), structural biology using AlphaFold, bioinformatics, and data science. Participants came primarily from Latin America (90%), with additional observers from the United States, Africa, and Asia. The KEM served as a platform to showcase research projects, case studies, and real-world applications of AI in pathogen research, human health, microbiomes, agriculture, bioethics, and epidemiology in the region. It also presented teaching and training strategies in AI. The event fostered the formation of a regional Community of Practice and curriculum-development taskforce of eight scientific leaders (principal investigators) who would later be responsible for developing and delivering the pilot course (module coordinators), who were selected based on their expertise in AI-based approaches, bioinformatics, data analysis, and bioscience, complemented by experience in teaching and scientific capacity building. Its composition also reflected the regional and interdisciplinary priorities identified during the KEM. The process additionally drew on the previous training experience of the UCR, CABANAnet and EMBL-EBI teams and the teaching experience of researchers from participating Latin American institutions. Details and KEM recordings at: https://sites.google.com/view/cabananetwork-kem/biotrain.

### 2.2 Development of the AI for biosciences course

Over 4 months, as previously indicated, the group of eight regional scientific leaders and experts in AI-based strategies (curriculum-development taskforce) was responsible for the design of the curriculum. To further strengthen this task, an open call for curriculum-development secondees (secondment program) attracted 26 applications. Secondees were defined as advanced PhD students, postdoctoral researchers, and early-career scientists with experience in relevant fields, providing them valuable opportunities to build international collaborations, expand professional networks, and strengthen and accelerate their scientific careers. Thus, for selection, a scoring system was applied to evaluate each application, based on the information provided, their AI skills, Latin American nationality, background in biosciences, and their potential for impact within the region. After this process, seven secondees (three women and four men) representing six Latin American countries (2 from Argentina, one from Bolivia, Brazil, Costa Rica, Mexico and Peru) were selected with a living expense allowance and supervised by an academic tutor (a member of the taskforce team) during their internship. Together, they jointly worked over approximately three months, contributing to the development and refinement of the curriculum and materials. This provided additional perspectives from researchers at different career stages and from diverse national and institutional contexts, which additionally served to seed and sustain the Community of Practice established as part of the BiotrAIn project.

Regarding the curriculum topics, learning objectives and contents were established through an iterative process combining the needs identified through the KEM, community survey (below), an assessment of existing AI and bioinformatics training programs (from academia), and the expertise of the curriculum-development and training teams. The process also incorporated established guidance on responsible AI and data management, including UNESCO recommendations and the FAIR principles promoted through EMBL-EBI activities. Regular discussions and feedback among the core team, taskforce leads, secondees, and the broader trainer group were used to refining the final content before and after training courses.

The resulting curriculum co-developed consisted of the learning outcomes, contents and high-quality, open-access training materials that formed the foundation of the course. It was designed to support the practical application of AI to biological data while enabling participants to subsequently share and teach these approaches within their own communities. The curriculum includes modules on: (i) General concepts of AI, (ii) Data pre-processing, (iii) Ethics and responsible AI use, (iv) Classification, (v) Clustering, (vi) Deep learning, (vii) AlphaFold for structural biology, (viii) Train-the-trainer session to further amplify the dissemination of the course materials. Importantly, the datasets selected for the training are from Latin American projects, amplifying the relevance of local science. The complete curriculum (including three versions) is available as an open resource on GitHub: https://github.com/orgs/BiotrAIn-community/repositories.

### 2.3 Training sessions

1. *Pilot course*: The curriculum was initially piloted through a five-day face-to-face course at the University of Costa Rica from 16 to 20 June 2025, with 25 participants (young researchers and postgraduate students), eight trainers, core team and most of the secondees. The general strategy was inspired by previous experiences with CABANAnet (Campos-Sánchez et al., 2024) and AlphaFold Education summit (Magaña et al., 2026). For participant selection, a scoring system was applied to evaluate each application. There were 118 applications for the 25 available places, with travel and accommodation support provided for selected participants. Applicants were evaluated based on basic familiarity with R, Python, or another programming language, intermediate English level, as well as a background in a relevant biological field. Applications were independently assessed by three members of the core team using the scoring instruments. Geographic representation, gender equity, and the inclusion of traditionally underrepresented groups were also considered during selection. The resulting cohort represented a broad range of Latin American countries and cities, including Argentina (Pergamino, La Plata, and Ciudad Autónoma de Buenos Aires), Bolivia (Cochabamba), Brazil (Ribeirão Preto, Belém, Belo Horizonte, São Paulo, and Piracicaba), Chile (Santiago), Colombia (Tunja), Costa Rica (San José), Mexico (Irapuato, Morelia, and Guanajuato), Paraguay (Asunción), Peru (Lima), and Uruguay (Canelones and Montevideo). This geographic distribution reflected the project's explicit intention to promote geographic diversity and inclusion. The course integrated all modules and offered practical applications in areas such as pathogen surveillance, biodiversity and ecology, microbiome analysis, and cancer research. In addition, the training included sessions on AI ethics and environmental considerations, as well as a dedicated Train-the-trainer track for participants who would subsequently teach and disseminate knowledge within their institutions or projects.
2. *Hub-and-spokes courses*: Updated versions of the course were conducted in a multi-country format (hub-and-spoke model) from 20 to 24 April 2026 across six Latin American countries, where central coordinating hub (Costa Rica) supported six local training nodes or “spokes,” enabling localized participation while fostering regional collaboration and expert exchange. The spokes were selected based on a call for six eligible host institutions, all of which were financially supported. Host applications were evaluated based on demonstrated impact and reach, feasibility of implementation, sustainability and long-term impact potential, and overall quality of the submission. The final list of spokes were Colombia, Mexico, Chile, Argentina, Brazil and Uruguay, completing seven classrooms (hub + spokes). Regarding trainees, demand for the Hub-and-Spokes course remained high, with 235 applications and 169 participants selected, that according to their residence includes 12 countries: Mexico, Colombia, Argentina, Uruguay, Chile, Brazil, Ecuador, Panama, Peru, Costa Rica, Bolivia, El Salvador. Participants were selected based on the same criteria as the pilot course. Across the seven classrooms, 57 trainers and classroom organizers supported course delivery. The model combined local classroom teaching with live cross-site Q&A and networking, a shared handbook, and a Slack workspace, facilitating interactions among participants across institutions and countries. The program was further strengthened through a multilingual webinar series and the development of promotional blog posts designed to increase visibility, accessibility, and engagement across diverse scientific communities in Latin America (https://www.ebi.ac.uk/training/events/biotrain-project-opportunities-latin-america/).
3. *BiotrAIn virtual course*: A subsequent virtual, 5-week, asynchronous course in August-September 2026, attracted 325 applications, of which 260 participants were accepted. In line with previous courses, participants the selection criteria were less restrictive, requiring only basic familiarity with a programming language, basic English level and a background in biological or related sciences. The refined materials were used, including recordings of the theoretical content and solved tutorials, which were implemented and evaluated in the previous phases. This broader approach was consistent with the objective of expanding access to the training materials to the scientific community, including students and professionals at all levels.

For all stages and courses, recruitment and dissemination of the project activities were promoted using the established scientific and training networks, including mailing lists associated with CABANAnet (>400 contacts) and the Laboratory of Bioinformatics at the University of Costa Rica (>750 contacts), in addition to social media and other communication channels.

## 3 Integration: regional AI state in biosciences assessment

Alongside the key activities (the KEM, course development, and training sessions), the BiotrAIn project conducted a comprehensive assessment of the current state of AI in life sciences in Latin America. This assessment included results from a survey conducted with 118 bioscientists across the region. A systematic analysis of the collected data was carried out to identify the main barriers, challenges, and opportunities related to AI development and adoption. Analysis based on the Ishikawa diagram (cause-effect) and Pareto Chart was applied to highlight priority challenges, their impact, and their relative importance. Results informed the BiotrAIn course and training session development.

As shown in Figures 2, 3, there are multiple factors affecting the development of AI in Latin America:

**Figure 2:**
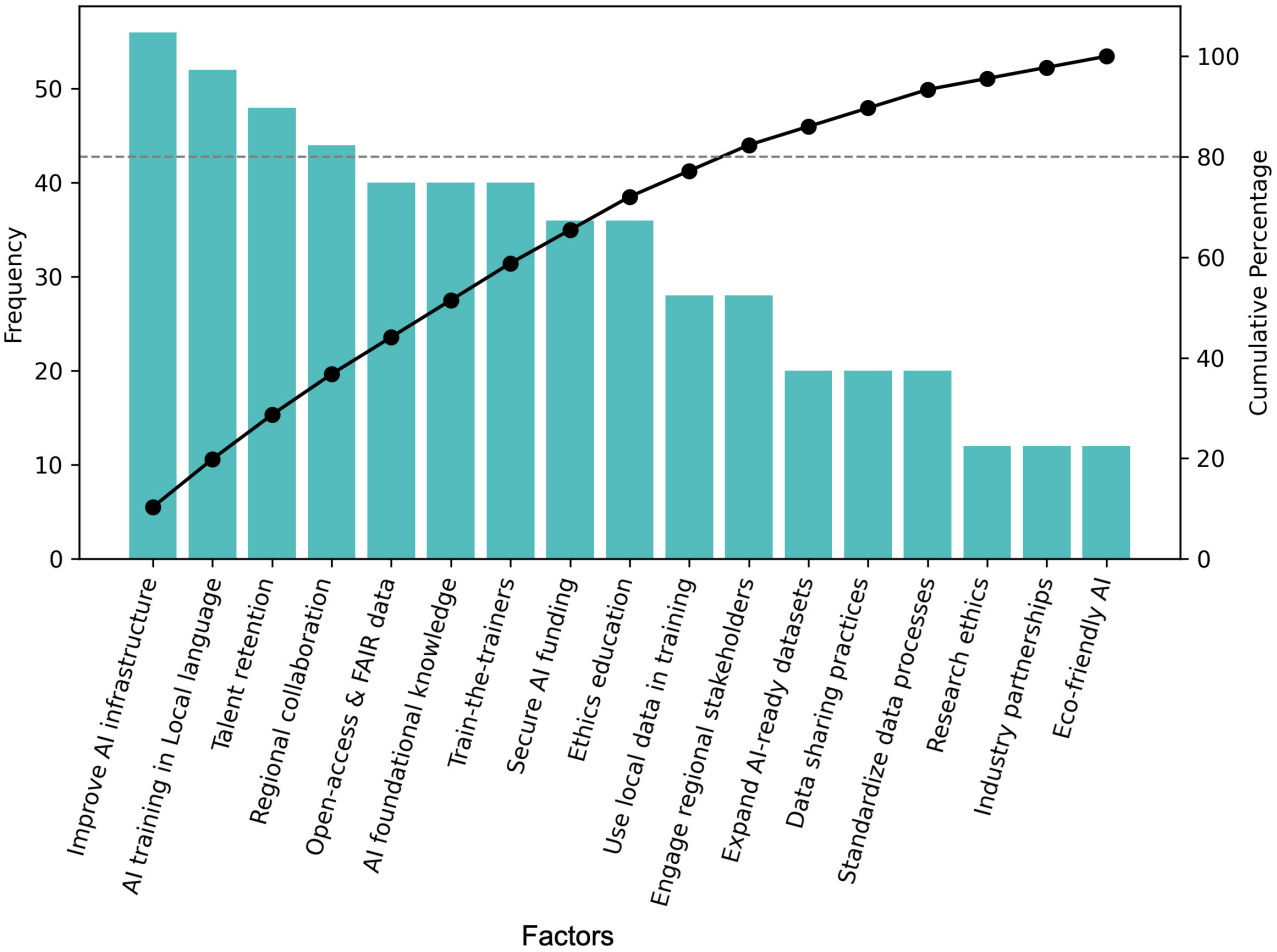
Pareto chart of the main factors affecting the development of AI in biosciences in Latin America based on the BiotrAIn survey completed by 118 bioscientists.

**Figure 3:**
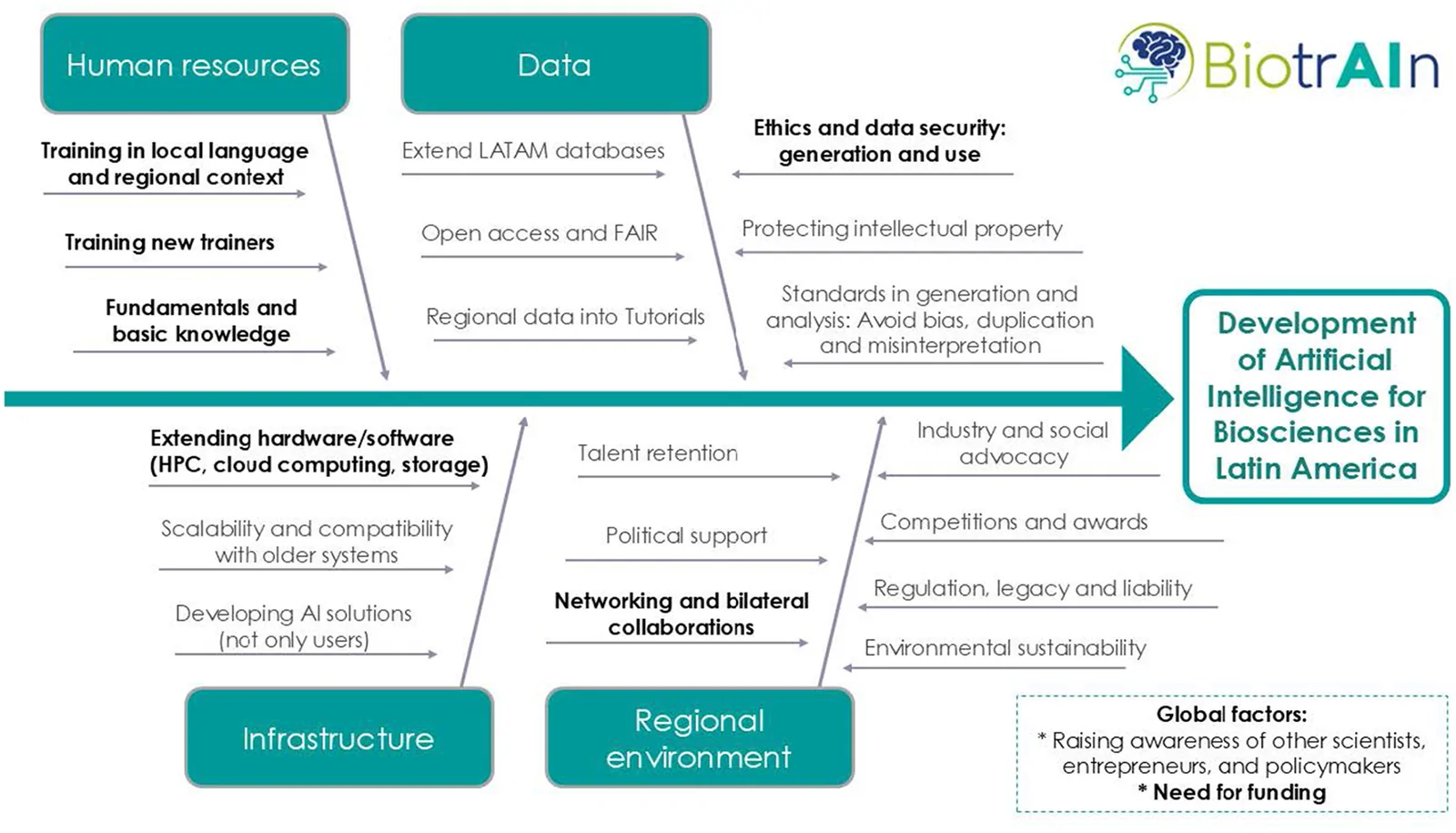
Ishikawa (cause-effect) diagram showing the main categories and factors hindering the development of AI in biosciences in Latin America collected in the BiotrAIn survey. Factors in bold were addressed by the BiotrAIn project.

1. *Infrastructure and resources:* The most frequently indicated barrier is the need to improve AI infrastructure across the region, including access to computing power, cloud platforms, and digital connectivity. This deficit is closely tied to insufficient and temporary funding mechanisms that limit institutions' ability to invest in long-term AI research, training, and implementation projects (Filgueiras, 2023). Without strategic investment and infrastructure upgrades, many countries in the region struggle to compete globally or even sustain local AI initiatives (Sussman et al., 2022).
2. *Human resources, capacity building, and education:* Another major factor limiting progress is the gap in education and capacity building tailored to the Latin American context. While there is a growing interest in AI, the availability of local training programs remains limited. These should reflect local languages, societal challenges, and resource constraints. Foundational AI knowledge must be more broadly integrated into academic and technical training pipelines, not only for students but also through “train-the-trainer” approaches that empower educators to scale teaching efforts (Fernández-Miranda et al., 2024). Strengthening human capital is closely linked to talent loss. The region continues to experience brain drain, as skilled professionals often leave in search of better opportunities abroad. Retaining this talent requires both better working conditions and opportunities for meaningful engagement with cutting-edge AI work locally (Campos-Sánchez et al., 2024).
3. *Regional integration and collaboration ecosystem:* Collaboration within the region is also crucial. Stronger regional networks and cooperation among academic institutions, governments, and the private sector are essential to overcoming the fragmentation that hampers innovation. Equally important is the need to use local data in AI model development. Many AI tools are currently based on data from the Global North, which undermines their accuracy and relevance for Latin American populations. Engaging regional stakeholders in data collection, governance, and application ensures AI systems are developed in ways that are both more representative and more equitable (Magaña et al., 2026; Rojas, 2025).
4. *Data access:* The ability to work with data (collecting, sharing, and using it effectively) is another fundamental issue. Open-access and FAIR (Findable, Accessible, Interoperable, Reusable) data principles are increasingly recognized as priorities, as they promote collaboration and reduce redundancy. However, challenges remain in standardizing data collection practices and encouraging data sharing across institutions and countries (Sussman et al., 2022). Expanding the availability of high-quality, ethically valid, AI-ready datasets tailored to local realities is key for both academic research and practical applications in health, education, agriculture, and other sectors (Molina-Mora and Herrera-Hidalgo, 2025).

Finally, although less frequently mentioned in the survey, emerging priorities indicate the need for stronger ethical frameworks, deeper integration with the private sector, and environmentally sustainable AI development (Filgueiras, 2023). Ethics education and governance are particularly important in a region with diverse cultural and legal norms, ensuring that AI is developed responsibly and equitably (Magaña et al., 2026; Rojas, 2025). Partnerships with industry can help bridge the gap between research and real-world implementation, while concerns about the environmental footprint of AI are beginning to influence discussions around sustainable development. This is discussed as follows.

### 3.1 Data governance and equity related to AI in Latin America

The development and adoption of AI in Latin America must also be considered within the broader context of data governance, responsible innovation, and equity (Wahl et al., 2026). AI systems depend on access to high-quality and appropriately governed data, yet countries and institutions across the region differ substantially in their data infrastructures, regulatory frameworks, computational resources, and technical expertise (Hussein et al., 2026). These differences can influence not only the capacity to develop and deploy AI systems, but also whose data, knowledge, and research priorities are represented in AI applications. Recent regional initiatives have therefore emphasized governance, data and infrastructure, and human capital as interconnected pillars for building sustainable and equitable AI ecosystems (Carvajal Piña and Vu, 2025; Pimenow et al., 2025).

Responsible AI in this context extends beyond technical performance to include privacy and data protection, transparency, accountability, human oversight, fairness, and the appropriate governance of data throughout its lifecycle (Papagiannidis et al., 2025). These considerations are particularly relevant for biosciences, where AI applications frequently involve sensitive human, health, genomic, or ecological data and may involve cross-institutional or cross-border data sharing. International frameworks such as the UNESCO Recommendation on the Ethics of AI emphasize inclusive and rights-based approaches to data governance, equity, non-discrimination, and capacity building (UNESCO, 2023). In Latin America, these principles are increasingly reflected in regional efforts to develop ethical and inclusive AI governance while strengthening local institutional capacities (UNESCO, 2026).

From this perspective, capacity building is an important component of responsible and equitable AI adoption. Differences in access to expertise, computational infrastructure, training opportunities, and locally relevant datasets may otherwise reinforce existing inequalities between countries and institutions (Wahl et al., 2026). The BiotrAIn project addresses part of this challenge by promoting open training resources, regional collaboration, local trainers, and practical skills that can be adapted to different research contexts. The BiotrAIn strategy employed in-depth biographical research and interactive activities involving students from different countries. The “Ethics” module encouraged them to investigate and critically reflect on how AI and governance issues are addressed in their respective countries, contributing significantly to the project's success. This approach fostered learning, critical reflection, and debate regarding the application of AI tools. These outcomes were further reinforced with several activities during the courses, which strengthened the students' ability to engage in discussions, build connections, and reflect on how AI can be applied across different contexts and countries.

However, training alone cannot resolve structural differences in infrastructure, data availability, institutional capacity, or governance. Sustainable progress will therefore require continued regional cooperation, investment in infrastructure and human capital, locally contextualized training, and governance approaches that ensure that the benefits of AI development are broadly accessible across Latin American research communities (Carvajal Piña and Vu, 2025).

## 4 BiotrAIn assessment

### 4.1 Strengths and wider outcomes

One of the main strengths of BiotrAIn was its distributed and collaborative approach to capacity strengthening. Rather than focusing exclusively on training participants, the project developed a regional Community of Practice and progressively expanded from a core team to a curriculum-development taskforce, a network of trainers and facilitators, and locally supported training sites. The curriculum-development secondment program also provided early-career researchers with an active role in developing training materials, while the Train-the-Trainer component was designed to support the subsequent dissemination of knowledge within local communities. This progression created an initial multiplier effect, with some participants from the pilot course subsequently contributing as trainers or facilitators in the Hub-and-Spokes program.

A second strength was the emphasis on practical and openly reusable resources. The curriculum covered a broad range of AI applications relevant to bioscience, including data preprocessing, clustering, classification, deep learning, AlphaFold, and responsible AI and ethics. Lecture materials, practical exercises, instructor guides and other resources were made openly available through GitHub under a CC BY-NC-SA 4.0 license, allowing their reuse and adaptation beyond the original project.

The project also demonstrated continued demand for AI training in the region. Beyond the initially scoped activities, the 5-week online BiotrAIn course attracted 325 participants from 17 countries, exceeding the number of participants trained through the pilot and Hub-and-Spokes activities combined. Continued dissemination through regional and international scientific events, together with further development of the training materials and community, provides evidence that the network and resources established through BiotrAIn can support activities beyond the original project period.

### 4.2 Limitations

Several limitations were also identified through participant feedback, discussions among the project team and trainers, and experience gained during implementation. First, the broad scope of the curriculum was both a strength and a limitation. The use of examples spanning pathogen genomics, human and animal health, biodiversity, agriculture, drug discovery and other areas allowed the materials to be relevant to diverse bioscience communities. However, this breadth also meant that the curriculum could not address the specific methodological and data needs of individual scientific disciplines in depth. Participants seeking to implement AI in a particular research area may therefore require additional, domain-specific training.

Second, the breadth and density of some modules presented challenges in terms of the time available for delivery. Some topics contained substantial theoretical and practical content, and feedback indicated that additional time could be beneficial for consolidation and hands-on activities. This limitation informed the design of subsequent online activities, in which participants were given longer periods to complete materials and additional activities were incorporated to accommodate different learning paces.

A further limitation concerns language. Although English was retained as the principal language of the curriculum to facilitate international collaboration and connection with the broader scientific and training communities, Latin America and the Caribbean are linguistically diverse, with Spanish and Portuguese predominating in much of the region and other local languages also being relevant. To partially address this challenge, the Hub-and-Spokes activities were delivered in the local language at participating sites, while the core resources remained in English. Nevertheless, language remains an important consideration for future regional capacity-building initiatives.

Finally, the rapidly evolving nature of AI represents a continuing challenge for curriculum development. For example, agent-based AI has expanded substantially since the project was initially designed and was not included as a dedicated component of the original curriculum. Maintaining the relevance of AI training therefore requires mechanisms for periodically updating both content and practical examples.

### 4.3 Lessons learned and generalizable implications

Several lessons from BiotrAIn may be relevant to future AI-training collaborations in Latin America and other regions. First, a distributed training model can be more sustainable than relying exclusively on a central training team. Developing local trainers and facilitators and involving them in curriculum adaptation can help establish capacity that remains within participating institutions after the end of a project.

Second, broad, openly accessible core materials can provide a useful foundation, but these should be designed to allow adaptation to specific scientific disciplines, languages and local contexts. The experience of BiotrAIn suggests that a regional curriculum should provide common AI and data-science competencies while allowing local trainers to introduce domain-specific datasets, examples and practical exercises.

Third, regional relevance is important not only in the choice of scientific examples but also in the datasets and research contexts used during training. Latin America is highly heterogeneous in terms of scientific infrastructure, socioeconomic conditions, ecological settings and research priorities. Future programs could therefore benefit from combining a common regional curriculum with country- or discipline-specific modules and datasets.

Finally, AI training should be considered an iterative process rather than a one-time intervention. Continuous feedback from participants and trainers, periodic updating of materials, and mechanisms for maintaining the Community of Practice can help ensure that training remains aligned with rapidly changing AI technologies and with evolving regional needs.

## 5 Conclusions

AI holds significant promise for advancing biosciences in Latin America, offering powerful tools for data analysis, disease modeling, and biological research. However, the region faces persistent challenges that limit its ability to fully harness this potential. These include limited infrastructure, fragmented research systems, underdeveloped training programs, and a lack of coordinated policy frameworks. Despite these obstacles, several initiatives and studies have shown that AI is being successfully applied in areas such as epidemiology, health, biodiversity, and agriculture, often using local data and tailored strategies. To advance further, the region must invest in capacity building, especially through localized training, and efforts to retain skilled professionals. Strengthening regional collaboration and ensuring data sovereignty are essential for building culturally relevant and ethically sound AI applications. Initiatives like BiotrAIn exemplify how targeted programs can help bridge the gap, fostering inclusive and sustainable innovation. We hope that this may serve as a model and set of open resources for other regions globally to adapt to their settings. Moving forward, strategic investment, cross-sector partnerships, and stronger policy support will be key to ensuring that Latin America not only adopts AI technologies but actively shapes their development for local and global benefit.

## Data Availability

The original contributions presented in the study are included in the article/Supplementary material, further inquiries can be directed to the corresponding authors.
