# Supplementary material for: Landscape and training opportunities in artificial intelligence for biosciences in Latin America: the BiotrAIn project
Source: Front Artif Intell. 2026 Oct 14;9:1931300. doi: 10.3389/frai.2026.1931300 (PMC13713524; doi:10.3389/frai.2026.1931300)
Supplement: Supplementary file 1 [file Data_Sheet_1.zip › Form 1 BiotrAIn secondment application - Formularios de Google.pdf]

# Application for a BiotrAIn secondment:

**Spanish version:** https://forms.gle/8mF6nduAntkiMH3YA

Calling all researchers based in Latin America! Use this form to apply for a BiotrAIn secondment (in-person, virtual, or a combination of the two), up to 3 months in length to develop training resources on AI for the biosciences. We welcome doctoral students, post-doctoral researchers and researchers at the MSc. level and above, based in Latin America, to seize this unique opportunity. We expect successful applicants to have at least basic experience of programming in R or Python. We anticipate secondments being fulltime - if this is not possible for you please contact us by email cabananet.network@gmail.com

**Duration:** up to 3 months
**Expected start date:** March/April 2025

**Expected end date:** May/June 2025

**Compensation:** $1000-1500 USD / month depending on location.

**Location:** Participating institutions in Latin America or at EMBL-EBI, UK

***General objective of the project***
Targeted training, by empowering bioscientists with essential AI skills, and promoting interdisciplinary, transnational and international collaboration.

***Specific objectives of the project***
Our goal is to create a fundamental and sustainable curriculum on AI for bioscientists from Latin America, including data science basics, and leading towards the use and application of AI to better solve biological and biomedical problems. Our intended impact is that Latin America is able to participate as an equal partner in globally important bioscience projects that use or develop artificial intelligence methods.

***Secondment Benefits***

- Engage and connect with the bioinformatics research community of CABANAnet and BiotrAIn.
- Collaborate with Latin American bioinformatics researchers in a different country (secondments must take place in a different country to your residing country).
- Develop training and course authoring skills.
- Living expense allowance (LEA) of 1000-1500 USD per month.

***Secondee Responsibilities***

- Ensure health insurance coverage while in the host institution.

- To develop context-relevant training content.
- Make the training materials developed is openly accessible with the support of the BiotrAIn project partners.
- Share knowledge in a workshop or meeting organized or supported by BiotrAIn (any reasonable costs will be covered by the BiotrAIn project).
- Submit monthly reports (written or oral) about the progress of the work.

Only fully completed forms will be considered. **Applicants must be from Latin America.** The **deadline for submissions is 3th March 2025**. Successful candidates will commence their secondment (initially virtually) by March/April 2025. To receive funding for your secondment, your country of residence must be on the DAC list of ODA recipients: http://www.oecd.org/dac/stats/documentupload/DAC%20List%20of%20ODA%20Recipients%202014%20final.pdf

* Indica que la pregunta es obligatoria

1. Your full name *

2. Your institutional email address *

3. Full legal name of the Institution where you are registered *

4. Country of residence *

*Marca solo un óvalo.*

- Argentina
- Brazil
- Colombia
- Costa Rica
- Mexico
- Peru
- Uruguay
- Chile
- Otros:

5. Highest qualification *

*Marca solo un óvalo.*

- Bachelor's degree
- Master's degree
- Doctoral degree
- Posdoctoral degree
- Otros:

6. Name of your supervisor (if you are a graduate student or postdoctoral researcher) or Head of Department or Institute (if you are an independent researcher) *

7. Are you available to start your secondment in March/April 2025? How early are you available to start? *

8. Please provide a letter of support, on headed paper, from your supervisor (graduate students and postdoctoral fellows) or your head of department (independent researchers). *

   Archivos enviados:

9. Please upload a short CV of no more than 2 pages, highlighting the skills that are especially relevant to this project, plus 1 page maximum of publications *

   Archivos enviados:

10. Will you be partially supported by your current research funds throughout your secondment? *

    *Marca solo un óvalo.*

    - Yes
    - No
    - I do not know
    - Otros:

11. Provide your goals for the project (100 words) *

12. Please confirm that you agree to the open publication of any outputs from your secondment. If you cannot agree to one or more of the checkbox statements, please use the 'other' field to provide a justification. *

*Selecciona todas las opciones que correspondan.*

- [ ] I agree to any research papers arising directly from my secondment project to be published in open-access journals.
- [ ] I agree that any software I develop as part of my secondment will be licensed under an open license
- [ ] I agree that any datasets generated directly through my research secondment will be made openly available through public data resources and tools
- [ ] I agree that any training materials generated as a direct result of my secondment will be given a CC-BY-SA license and made available through EMBL-EBI's Train online portal
- [ ] Otros: ____________________

13. How does the study support your future research and career objectives? What new skills do you intend to learn during the secondment? (200 words) *

14. How does your secondment support and impact on the research and teaching objectives of your current institution(s)? How will you share what you have learned with your colleagues? (200 words) *

15. What opportunities are available to deliver the training that you develop at your home institute, expand your research interests, foster collaborative links, and secure research funding after your secondment? How will this secondment enhance those opportunities and how do you propose to contribute to strengthening your local research networks? (200 words) *

## Additional information about yourself

In this section we ask you some questions about yourself. BiotrAIn is committed to doing everything it can to support scientists from underrepresented groups who would not otherwise have access to opportunities with the international research community. We need to ask some questions about your citizenship and any under-represented group with which you most closely identify so that we can honor this commitment and so that we can track our impact on scientists from underrepresented groups.

16. What is your gender

*Marca solo un óvalo.*

- Female
- Male
- Non-binary
- Prefer not to say
- Otros:

17. What is your Nationality

18. What is your Country of Citizenship

19. Please state any under-represented group that you identify with.

This includes nationality, ethnic group, indigenous groups, or any historically marginalized group.

## Consent

20. Do you consent the BiotrAIN project to use your anonymized information provided in this form to be used for analysis and to create a database of contacts? *

*Marca solo un óvalo.*

- Yes
- No

## Thank you for your application!

Deadline for Applications: 3 March 2025

Google no creó ni aprobó este contenido.

Google Formularios
