# Supplementary material for: Landscape and training opportunities in artificial intelligence for biosciences in Latin America: the BiotrAIn project
Source: Front Artif Intell. 2026 Oct 14;9:1931300. doi: 10.3389/frai.2026.1931300 (PMC13713524; doi:10.3389/frai.2026.1931300)
Supplement: Supplementary file 1 [file Data_Sheet_1.zip › Form 2 - Pilot Course BiotrAIn course_ Concepts, approaches and applications of Artificial Intelligence in bioscience - Formularios de Google.pdf]

BiotrAIn course: Concepts, approaches and applications of Artificial Intelligence in bioscience

This course on Artificial Intelligence (AI) for the biosciences is hybridin-person and will take place at the Continuing Education Building of the University of Costa Rica (UCR), San José Costa Rica. It will include a computer laboratory component and a data analysis section. It will not be possible to participate in the course remotely. Places are limited.
It is intended for students, researchers, and professionals in biological sciences from Latin America.

Pre-requisites: We assume participants will have basic level experience with Python and R to attend the course.

Program:
The course comprises 5 daily modules with fundamentals, applications and tutorials for the following topics:
(i) Module 1: Introduction to AI (concepts, bioethics and data management),
(ii) Module 2: clustering,
(iii) Module 3: classification,
(iv) Module 4: deep learning, and
(v) Module 5: Train-the-trainer.

Learning objectives:
By the end of this course, participants will be able to apply foundational concepts of artificial intelligence and machine learning to biological data, including situations in the LATAM context, leveraging their existing data analysis skills to:
Module 1: Identify appropriate AI concepts and techniques, ethical aspects including responsible AI, and general usability of AI in bioscience.
Module 2: Preprocess and analyze biological datasets using clustering algorithms, including regional databases (LATAM).
Module 3: Build and evaluate classification models to address biological questions in the LATAM context.
Module 4: Apply deep learning models, including tools such as AlphaFold, to analyze complex biological research problems.
Module 5: To deliver AI contents with best teaching/learning practices in biological research.

Dates: June 16-20th, 2025
Application deadline: April 7th, 11:59 PM (UTC-6)
Selection notification: April 11th

Language: English. Please note that the course delivery and materials will be in English. We expect at least intermediate level English to participate.

Scholarships: All participants will receive scholarships to attend the course. The scholarship includes airfare (a maximum amount per route will be provided, including a carry-on bag), four nights of accommodation in a shared room with another course participant, and meals (breakfast, lunch, and two snacks).

Not covered: Ground transportation, dinners, extra travel expenses, visa to Costa Rica (check the requirements for your country) and life insurance (having life insurance is a requirement to participate in the course).
Contact: cabananet.network@gmail.com

Organized by: BiotrAIn project (https://www.ebi.ac.uk/training/our-partnerships/biotrain), UCR, EMBL's European Bioinformatics Institute (EMBL-EBI).

* Indica que la pregunta es obligatoria

1. Write your full name. *

____

2. Write your identification number (ID card, passport, or residency number). *

____

3. Write your email address. *

____

4. What is your gender? *

Marca solo un óvalo.

- Female
- Male
- Non-binary
- Prefer not to say
- Other

5. What is your Nationality? *

____

6. What is your country of residence? E.g., Costa Rica. *

____

7. What is your city of residence? E.g., Bogotá. *

____

8. Please state any under-represented group that you identify with. *

This includes nationality, ethnic group, indigenous groups, or any historically marginalized group.

____

9. What is your field of academic training? E.g., biology, microbiology. *

____

10. Highest qualification *

Marca solo un óvalo.

- Bachelor's degree
- Master's degree
- Doctoral degree
- Posdoctoral degree
- Other

11. Do you work or study? *

Marca solo un óvalo.

- Work
- Study
- Both
- Neither

12. Name the institution where you study or work. Please write the full name, without abbreviations. *

____

13. What is your level of proficiency in the English language? *

Marca solo un óvalo por fila.

	None	Basic	Intermediate	Advanced
**Reading**	⬭	⬭	⬭	⬭
**Listening**	⬭	⬭	⬭	⬭
**Speaking**	⬭	⬭	⬭	⬭

14. What is your level of proficiency in the following computing and statistical skills? *

Marca solo un óvalo por fila.

	None	Basic	Intermediate	Advanced
**R programming**	⬭	⬭	⬭	⬭
**Unix**	⬭	⬭	⬭	⬭
**Python**	⬭	⬭	⬭	⬭
**Statistical analysis**	⬭	⬭	⬭	⬭

15. Do you have experience using AI in your work? It could be for research, teaching or other applications. *

16. How does attending this course supports your future research and career objectives? What new skills do you intend to learn during this course? (maximum 200 words). *

17. Do you need a visa to visit Costa Rica? *

Marca solo un óvalo.

- Yes
- No

18. Please upload a short CV of no more than 2 pages, highlighting the skills and experience that are especially relevant to this course, plus 1 page maximum of publications *

Archivos enviados:

19. Please provide a letter of support, on headed paper, from your supervisor (graduate students and postdoctoral fellows) or your head of department (independent researchers) for you to attend this course. *

Archivos enviados:

20. Do you anticipate being able to apply what you have learnt to teach others? *

21. Please write any comments, clarifications regarding any previous questions, or any questions you may have. *

Consent

22. Do you consent to the BiotrAIn project using your anonymized information provided in this form for analysis and to create a database of contacts? *

Marca solo un óvalo.

- Yes
- No

Thank you for your application!

Remember to press SUBMIT to complete the process.

BiotrAIn team

Google no creó ni aprobó este contenido.

Google Formularios
