# Supplementary material for: Landscape and training opportunities in artificial intelligence for biosciences in Latin America: the BiotrAIn project
Source: Front Artif Intell. 2026 Oct 14;9:1931300. doi: 10.3389/frai.2026.1931300 (PMC13713524; doi:10.3389/frai.2026.1931300)
Supplement: Supplementary file 1 [file Data_Sheet_1.zip › Form 3 - Hub&Spokes HOSTs - Application form - BiotrAIn Hub and Spokes Course (hosts) - Formularios de Google.pdf]

Application form - BiotrAIn Hub and Spokes Course (hosts): Concepts, Approaches, and Applications of Artificial Intelligence in Bioscience

In response to the success of the BiotrAIn pilot course in June 2025, we are now looking for collaborators to run the next iteration of the course, which will be a distributed effort involving a 'hub' and multiple classrooms. By completing this form, you are expressing interest in hosting a classroom. This involves organising and hosting a BiotrAIn training workshop at a national or regional level, while connecting via videoconference with other participating classrooms across Latin America. This 'hub and spokes' model allows for local community engagement whilst also providing the trainees access to expertise from throughout Latin America scientific network.

Application Process: Please complete this form with details about your proposed activity (workshop or training session).
Application deadline: September 15th, 2025, 23:59 (UTC-6)
Selection criteria: Applications will be evaluated based on their demonstrated impact and reach, feasibility of implementation, sustainability and long-term impact potential, and overall quality of the application submission. Successful applicants will be notified by **30 September 2025**.
Late applications will not be considered for this funding round. Future funding rounds may be announced later, but we recommend applying as soon as possible for this round.
Prerequisites: please familiarise yourself with the course materials (please note this is version 1 of the materials, for context, the materials will be updated for the Hub and Spokes course): https://github.com/biotrain-latam/BiotrAIn-pilot-course

We especially encourage participants and trainers from the BiotrAIn pilot course to apply.

If you have any questions, please email **cabananet.network@gmail.com** with the subject header: **BiotrAIn - Hub and Spokes Course**

* Indica que la pregunta es obligatoria

1. Correo electrónico *

FAQ / Guidance

Q: What costs can the workshop funding cover?

A: Funding can be used for venue hire, catering, and travel expenses for speakers (where essential) or participants, as well as any essential requirements to run the event (e.g., AV equipment). Administrative costs (overheads) must also be included within the budget limit specified and must not be greater than 15% (as per Chan Zuckerberg Initiative rules).

Q: Can funding be used for stipends or salary costs?
A: Funding may be considered for stipends to support specific, short-term tasks directly related to the workshop. However, funding for ongoing salary costs is not supported.

Q: What is meant by underrepresented groups?
A: This includes, but is not limited to, women, ethnic minorities, researchers with disabilities, early-career researchers, researchers from low-resource settings, and researchers experiencing barriers to access.

Q: Are virtual or hybrid workshops eligible for funding?
A: All the workshops need to be hybrid - all participants need to attend in person in your classroom, but you also need to make provision to connect to other classrooms remotely (this is the hub and spokes model). Please provide detailed information about the online platform and any physical venue involved.

Q: What is expected regarding the data sharing after the workshop?
A: We will provide a standardised feedback form to collect basic aggregated, anonymised impact measures (e.g., attendee numbers, participant feedback, knowledge gain metrics and a brief narrative report). This data will help us track the impact of the events we fund.

Q: Can I submit more than one application?
A: We try to maximise the reach of BiotrAIn workshops and ensure equitable distribution of funding. An Institution and the lead applicant can only submit one application; however individuals from that institution might feature as guest trainers in other applications.

Q: What type of follow-up activities are considered for sustainability and long-term impact?
A: Examples include train-the-trainer (to further increase capacity across the region), follow-on workshops on specific aspects of the curriculum in further depth creating online resources and recording sessions where relevant, continuing to engage with the online forums created during the hub and spokes course to keep the community going, activities that build a supportive network and facilitate ongoing mentorship among participants, or planning future workshops. Furthermore, demonstrating a plan for the ongoing ownership and maintenance of additional content is highly valued. This includes ensuring that the content remains up-to-date and in sync with the core BiotrAIn content.

Applicant Information

2. Country/Region/City *

3. First name *

4. Last name *

5. Institution *

6. Current role / Position *

Marca solo un óvalo.

- Principal investigator
- Professor-Lecturer
- Postdoctoral researcher
- Staff scientist
- Research technician
- PhD student
- Master's student
- Undergraduate student
- Otros:

7. Email Address *

8. Team members (name, email, institution) *

Workshop/Training Session Details

9. We expect all classrooms to cover all modules of the course. Please indicate which of the below modules you have trainers for (we will provide the learning materials and can provide support with virtual trainers if needed, see next question): *

Selecciona todas las opciones que correspondan.

- [ ] Introduction
- [ ] Data pre-processing (new)
- [ ] Ethics
- [ ] Clustering
- [ ] Classification
- [ ] Deep Learning
- [ ] Alphafold
- [ ] Train-the-trainer
- [ ] None
- [ ] Otros: ____________________

10. Of the following modules, would you need support with trainers for (please select all that apply): *

Selecciona todas las opciones que correspondan.

- [ ] Introduction
- [ ] Data pre-processing (new)
- [ ] Ethics
- [ ] Clustering
- [ ] Classification
- [ ] Deep Learning
- [ ] Alphafold
- [ ] Train-the-trainer
- [ ] None
- [ ] Otros: ______________________

11. Anticipated number of participants (we suggest between 15-30 per classroom) *

____

12. Where do you anticipate the majority of your attendees will be based? *

____

13. Training venue information: *
Venue name, Address, City, Country, Accesibility features

____
____
____
____
____

14. Preferred date for hosting the workshop? We will go with the majority date from successful applicants. Please select all that apply. *

Selecciona todas las opciones que correspondan.

- [ ] 13 April - 17 April 2026
- [ ] 20 April - 24 April 2026
- [ ] Otros: ______________________

15. Total funding requested in US$ (attach your budget). Maximum US$14,000.00 per application. Please note, administrative costs (overheads) must also be included within the budget limit specified and must not be greater than 15% (as per Chan Zuckerberg Initiative rules). *

Archivos enviados:

16. Have you delivered this or a similar workshop before? *

Marca solo un óvalo.

( ) Yes

( ) No

17. If yes, provide details, links, programme info, and impact metrics (if you were not involved previously with BiotrAIn)

____

____

____

____

____

18. How do you plan to raise awareness and promote applications, particularly from underrepresented groups? *

19. Institutional events organiser contact: (Include names, contact details, and roles) *

20. Are you currently collaborating or working with another group/institute to deliver this workshop? *

Marca solo un óvalo.

- Yes
- No
- Otros:

21. If yes, please state which partner(s):
(Include full institution name and location)

22. Do you have any existing grants or institutional funding allocated for the workshop/training? *

Marca solo un óvalo.

- Yes
- No
- Pending

23. If yes or pending, specify the funding source and amount in US$.

____

24. Language of the course delivery in your classroom? *

Please note that course material are in English. The joint sessions and online communication will be in English

Marca solo un óvalo.

- Spanish
- Portuguese
- English

25. **Support needed:**

Describe the level of support required (e.g., help identifying speakers, promotion, tools for registration/delivery, guidance on structure, etc.)

26. Agreement to share anonymised impact data: *
(Condition of funding)

Marca solo un óvalo.

- Yes
- No

27. **Sustainability and long-term impact:** *

Describe how you plan to ensure the workshop's sustainability, including follow-up activities, future workshops, mentorship networks, or content updates in coordination with BiotrAIn content.

28. **Supporting Documents (Optional)**

You may upload documents such as promotional strategies.

Archivos enviados:

Thank you

Thank you for your application! Your proposal will be reviewed, and we will contact you soon regarding the outcome.

For any inquiries, please reach out to cabananet.network@gmail.com with the subject header **BiotrAIn - Hub and Spokes Course**

Best regards,
BiotrAIn Team

Google no creó ni aprobó este contenido.

Google Formularios
