# Supplementary material for: Landscape and training opportunities in artificial intelligence for biosciences in Latin America: the BiotrAIn project
Source: Front Artif Intell. 2026 Oct 14;9:1931300. doi: 10.3389/frai.2026.1931300 (PMC13713524; doi:10.3389/frai.2026.1931300)
Supplement: Supplementary file 1 [file Data_Sheet_1.zip › Form 4 - Hub&Spokes Participants - Application form - BiotrAIn Hub and Spokes Course (hosts) - Formularios de Google.pdf]

BiotrAIn Hub and Spokes Course (participants): Concepts, approaches and applications of Artificial Intelligence in bioscience

This course on Artificial Intelligence (AI) for the biosciences is in-person and will take place simultaneously at the the following sites:

- University of Buenos Aires (FCEN-UBA), Argentina
- CIFASIS-CONICET/UNR, Rosario, Argentina
- Aggeu Magalhães Institute / Fiocruz Pernambuco, Brazil
- ENES Unidad León, National Autonomous University of Mexico, León, Guanajuato, Mexico
- Universidad de Antioquia, Medellín, Colombia
- University Andres Bello, Santiago, Chile
- University of Costa Rica, San José, Costa Rica

It will include a computer laboratory component and a data analysis section. Remote participation will not be possible. Places are limited.
It is intended for students, researchers, and professionals in biological sciences from Latin America.

Pre-requisites: We require participants to demonstrate that they have basic level experience with Python and R to attend the course.

Program:
The course comprises 5 daily modules with fundamentals, applications and tutorials for the following topics:
(i) Module 1: Introduction to AI (concepts, bioethics and data management),
(ii) Module 2: clustering,
(iii) Module 3: classification,
(iv) Module 4: deep learning,
(v) Module 5: Train-the-trainer.

Learning objectives:
By the end of this course, participants will be able to apply foundational concepts of artificial intelligence and machine learning to biological data, including situations in the LATAM context, leveraging their existing data analysis skills to:
Module 1: Identify appropriate AI concepts and techniques, ethical aspects including responsible AI, and general usability of AI in bioscience.

Module 2: Preprocess and analyze biological datasets using clustering algorithms, including regional databases (LATAM).
Module 3: Build and evaluate classification models to address biological questions in the LATAM context.
Module 4: Apply deep learning models, including tools such as AlphaFold, to analyze complex biological research problems.
Module 5: To deliver AI contents with best teaching/learning practices in biological research.

Course dates: 20 - 24 April, 2026

Application deadline: 28 November, 2025, 23:59 (UTC-6)

Selection notification: 15 December, 2025

Language of course delivery: Spanish, Portuguese, English - please see the FAQ below for more information

Language of application, written communication and course materials: English only

Contact: If you have any questions, please email cabananet.network@gmail.com with the subject header: **BiotrAIn - Hub and Spokes Course**

* Indica que la pregunta es obligatoria

FAQ

Q: Can I apply to a classroom that is not in my region

Yes, you can apply to any of classrooms listed above, however we recommend applying to classrooms that are easy to reach from your region.

Q: Can I apply to be considered to more than one classroom

Yes, please indicate up to 2 classroom locations where you wish to be considered.

Q: Can I apply to join the classrooms online?

There's no provision for online attendance, the BiotrAIn hub and spokes course will be delivered in person at different physical classroom locations as indicated above.

Q: What languages will the course be delivered in ?

All course communication, including interactive Q&A sessions and course materials, will be in English. However, for the in-person sessions within each classroom, the live sessions of the course will be delivered in different languages across the multiple physical locations as indicated below:

- **English** - ENES Unidad León, National Autonomous University of Mexico, León, Guanajuato, Mexico.
- **Spanish** - University Andres Bello, Santiago, Chile; CIFASIS-CONICET/UNR, Rosario, Argentina; University of Costa Rica, San Jose, Costa Rica; University of Buenos Aires (FCEN-UBA), Argentina; Universidad de Antioquia, Medellín, Colombia.
- **Portuguese** - Aggeu Magalhães Institute / Fiocruz Pernambuco, Brazil

Q: What is meant by underrepresented groups?

A: This includes, but is not limited to, women, ethnic minorities, researchers with disabilities, early-career researchers, researchers from low-resource settings, and researchers experiencing barriers to access.

Q: What support is available to support course attendance

Funding to support local travel and accommodation is limited and funding availability varies by location.

Q: Question about accreditation: Will I receive academic credits?

Academic credit may vary by classroom. All participants who attend the entire course will receive a certificate of attendance.

Not covered: Ground transportation, dinners, extra travel expenses, visas (check the requirements for your country) and life insurance (having life insurance is a requirement to participate in the

course).

Personal Information

1. Write your full name. *

2. Write your email address. *

3. What is your gender? *

Marca solo un óvalo.

- Female
- Male
- Non-binary
- Prefer not to say
- Other

4. What is your Nationality? *

5. What is your country of residence? E.g., Costa Rica. *

6. What is your city of residence? E.g., Bogotá. *

7. Preferred Classroom Location 1 *

Marca solo un óvalo.

- University of Buenos Aires (FCEN-UBA), Argentina
- CIFASIS-CONICET/UNR, Rosario, Argentina
- Aggeu Magalhães Institute / Fiocruz Pernambuco, Brazil
- ENES Unidad León, National Autonomous University of Mexico, León, Guanajuato, Mexico
- Universidad de Antioquia, Medellín, Colombia
- University Andres Bello, Santiago, Chile
- University of Costa Rica

8. Preferred Classroom Location 2 *

Marca solo un óvalo.

- University of Buenos Aires (FCEN-UBA), Argentina
- CIFASIS-CONICET/UNR, Rosario, Argentina
- Aggeu Magalhães Institute / Fiocruz Pernambuco, Brazil
- ENES Unidad León, National Autonomous University of Mexico, León, Guanajuato, Mexico
- Universidad de Antioquia, Medellín, Colombia
- University Andres Bello, Santiago, Chile
- University of Costa Rica

9. Where did you learn about this course? *

10. Do you identify with any underrepresented groups in your field? *

11. If your application is successful, do you have any accessibility requirements we should be aware of in advance to support your attendance? (This will not be shown to the reviewers or affect your application result in any way) *

12. What financial support are you requesting. Please describe any financial support you need to attend this course. (Only applicable in at specific sites where support is needed). *
-Accommodation
-Travel

Academic and Professional Background

13. What is your field of academic training? E.g., biology, microbiology. *

14. Current role/position *

Marca solo un óvalo.

- Principal investigator
- Professor-Lecturer
- Postdoctoral researcher
- Staff scientist
- Research technician
- PhD student
- Master’s student
- Bachelor's degree
- Undergraduate student
- Otros:

15. Name the institution where you study or work. Please write the full name, without abbreviations. *

16. Department / Faculty *

17. What is your level of proficiency in the English language? *

Marca solo un óvalo por fila.

	None	Basic	Intermediate	Advanced
**Reading**				
**Listening**				
**Speaking**				

18. What is your level of proficiency in the following computing and statistical skills? *

Marca solo un óvalo por fila.

	None	Basic	Intermediate	Advanced
**R programming**				
**Unix**				
**Python**				
**Statistical analysis**				

19. Do you have experience using AI in your work? It could be for research, teaching or other applications. *

20. Briefly describe your current work. *

21. How does attending this course support your current work and career objectives? (Maximum 200 words) *

22. How will this course benefit the work of your group? *

Supporting Documents

23. **Curriculum Vitae (CV) - Maximum 2 pages plus 1 page of publications** *
Please upload a short CV of no more than 2 pages, highlighting the skills and experience that are especially relevant to this course, plus 1 page maximum of publications

Archivos enviados:

24. **Letter of Support from supervisor or head of department** *

Please provide a letter of support, on headed paper, from your supervisor (graduate students and postdoctoral fellows) or your head of department (independent researchers) for you to attend this course. Please ensure your letter of support includes the following; need for financial support and proficiency in python and R.

Archivos enviados:

Consent

25. Do you consent to the BiotrAIn project using your anonymized information provided in this form for analysis and to create a database of contacts? *

Marca solo un óvalo.

- Yes
- No

Thank you for your application!

Remember to press SUBMIT to complete the process.

BiotrAIn team

Google no creó ni aprobó este contenido.

Google Formularios
