# Supplementary material for: Landscape and training opportunities in artificial intelligence for biosciences in Latin America: the BiotrAIn project
Source: Front Artif Intell. 2026 Oct 14;9:1931300. doi: 10.3389/frai.2026.1931300 (PMC13713524; doi:10.3389/frai.2026.1931300)
Supplement: Supplementary file 1 [file Data_Sheet_1.zip › Form 5 - Virtual BiotrAIn Course Application - Formularios de Google.pdf]

Virtual BiotrAIn Course: Concepts, approaches and applications of Artificial Intelligence in bioscience

This course on Artificial Intelligence (AI) for the biosciences will be delivered fully online, with primarily offline (self-paced) learning activities, with two live synchronous sessions (one at the beginning and one at the end of the course). Participants who are unable to attend these sessions will have access to the corresponding recordings. Before completing this form, please review the course information available at the following link: https://drive.google.com/file/d/1fi8ObyXYGgBThygly1U9322Zx1nbjnZw/view?usp=sharing .

It is intended for graduate students, researchers, and professionals in biological sciences from Latin America.
Pre-requisites: We require graduate students, professionals and researchers to demonstrate that they have basic level experience with R and Python.

Program:
The course comprises 5 modules with fundamentals, applications and tutorials for the following topics:
(i) Module 1: Introduction to AI (concepts and data management),
(ii) Module 2: clustering,
(iii) Module 3: classification,
(iv) Module 4: deep learning,
(v) Module 5: Train-the-trainer and Ethics

Learning objectives:
By the end of this course, participants will be able to apply foundational concepts of artificial intelligence and machine learning to biological data, including situations in the LATAM context, leveraging their existing data analysis skills to:
Module 1: Identify appropriate AI concepts and techniques, ethical aspects including responsible AI, and general usability of AI in bioscience.
Module 2: Preprocess and analyze biological datasets using clustering algorithms, including regional databases (LATAM).
Module 3: Build and evaluate classification models to address biological questions in the LATAM context.
Module 4: Apply deep learning models, including tools such as AlphaFold, to analyze complex biological research problems.
Module 5: To deliver AI contents with best teaching/learning practices in biological research. Ethics is also integrated for responsible use of AI.

Course dates: August 17 - September 20, 2026

Application deadline: 30 July, 2026, 23:59 (UTC-6)

Selection notification: 7 August, 2026

Language of course delivery: English

Language of application, written communication and course materials: English only

Contact: If you have any questions, please email cabananet.network@gmail.com with the subject header: **BiotrAIn course**

* Indica que la pregunta es obligatoria

Personal Information

1. Write your full name. *

2. Write your email address. *

3. What is your gender? *

Marca solo un óvalo.

- Female
- Male
- Non-binary
- Prefer not to say
- Other

4. What is your Nationality? *

5. What is your country of residence? E.g., Costa Rica. *

6. What is your city of residence? E.g., Bogotá. *

7. Where did you learn about this course? *

8. Do you identify with any underrepresented groups in your field? *

This includes, but is not limited to, women, ethnic minorities, researchers with disabilities, early-career researchers, researchers from low-resource settings, and researchers experiencing barriers to access.

Academic and Professional Background

9. What is your field of academic training? E.g., biology, microbiology. *

10. Current role/position *

Marca solo un óvalo.

- Principal investigator
- Professor-Lecturer
- Postdoctoral researcher
- Staff scientist
- Research technician
- PhD student
- Master’s student
- Bachelor's degree
- Undergraduate student
- Otros: ______

11. Name the institution where you study or work. Please write the full name, without abbreviations. *

______

12. Department / Faculty *

______

13. What is your level of proficiency in the English language? *

Marca solo un óvalo por fila.

	None	Basic	Intermediate	Advanced
**Reading**	◯	◯	◯	◯
**Listening**	◯	◯	◯	◯
**Speaking**	◯	◯	◯	◯

14. What is your level of proficiency in the following computing and statistical skills? *

Marca solo un óvalo por fila.

	None	Basic	Intermediate	Advanced
**R programming**	◯	◯	◯	◯
**Unix**	◯	◯	◯	◯
**Python**	◯	◯	◯	◯
**Statistical analysis**	◯	◯	◯	◯

15. Do you have experience using AI in your work? It could be for research, teaching or other applications. *

Consent

16. Do you consent to the BiotrAIn project using your anonymized information provided in this form for analysis and to create a database of contacts? *

Marca solo un óvalo.

- Yes
- No

Thank you for your application!

Remember to press SUBMIT to complete the process.

BiotrAIn team

Google no creó ni aprobó este contenido.

Google Formularios
